# Increasing trends of anaphylaxis-related events: an analysis of anaphylaxis using nationwide data in Taiwan, 2001–2013

**DOI:** 10.1186/s40413-018-0202-7

**Published:** 2018-10-10

**Authors:** Tsung-Chieh Yao, Ann Chen Wu, Ya-Wen Huang, Jiu-Yao Wang, Hui-Ju Tsai

**Affiliations:** 1 Division of Allergy, Asthma, and Rheumatology, Department of Pediatrics, Chang Gung Memorial Hospital and Chang Gung University College of Medicine, Taoyuan, Taiwan; 2 Chang Gung Immunology Consortium, Chang Gung Memorial Hospital and Chang Gung University College of Medicine, Taoyuan, Taiwan; 3 Community Medicine Research Center, Chang Gung Memorial Hospital, Keelung, Taiwan; 4 PrecisiOn Medicine and Translational Research (PROMoTeR) Center, Department of Population Medicine, Harvard Pilgrim Health Care Institute and Harvard Medical School, Boston, MA USA; 5 Department of Pediatrics, Children’s Hospital, Boston, MA USA; 6 Division of Biostatistics and Bioinformatics, Institute of Population Health Sciences, National Health Research Institutes, Zhunan, Miaoli 350 Taiwan; 7 Department of Pediatrics, College of Medicine, National Cheng Kung University, Tainan, Taiwan; 8 Allergy and Clinical Immunology Research (ACIR) Centre, National Cheng Kung University, Tainan, Taiwan

**Keywords:** Anaphylaxis, Epidemiology, Incidence, Time trends, Asian

## Abstract

**Background:**

Anaphylaxis is a severe, potentially fatal, and systemic allergic reaction. Previous studies document increasing trends in incidence rates of anaphylaxis-related events in Western countries, yet little is known about the incidence and trend of anaphylaxis in Asia. In this study, we aimed to determine time trends in incidence rates of anaphylaxis-related events in Taiwan from 2001 through 2013.

**Methods:**

We utilized medical claims data from the National Health Insurance Research Databases in Taiwan. We identified anaphylaxis-related events (ICD-9-CM-codes: 995.0, 995.60–995.69, 999.41–999.42, and 999.49) and calculated incidence rates. Poisson regression models were applied to examine trends and incidence rates.

**Results:**

A total of 2496 patients (mean age, 45.11 years; 56% male) with first-time anaphylaxis were identified during 34,430,000 person-years of observation time. The overall incidence of anaphylaxis was 7.25 (95% confidence interval (CI) = 6.97–7.53) per 100,000 person-years, increasing from 4.79 in 2001 to 8.20 in 2013, with an incidence rate ratio (IRR) of 1.05 (95%CI = 1.04–1.06). Over the 13-year period, the increasing trends were found in incident diagnosis of anaphylaxis-related outpatient or emergency department visits (IRR = 1.06, 95%CI = 1.05–1.08) and admissions to intensive care units (IRR = 1.06, 95%CI = 1.03–1.10), whereas the trends in incidence of anaphylaxis-related hospitalizations remained steady. The proportion of patients requiring hospitalizations among all patients with anaphylaxis (*p*__trend_ = 0.01), as well as the proportion requiring intensive care treatment among patients who were hospitalized (*p*__trend_ = 0.01), both increased with age.

**Conclusion:**

The incidence rate of anaphylaxis in Taiwan has increased at an average rate of 5% annually since 2001, paralleling the rising trends in several Western countries.

**Electronic supplementary material:**

The online version of this article (10.1186/s40413-018-0202-7) contains supplementary material, which is available to authorized users.

## Background

Anaphylaxis, a severe and potentially fatal systemic reaction that is triggered suddenly by exposure to specific allergen substance, has been referred to as “the latest allergy epidemic” [1–3]. Previous studies have suggested an increase in the incidence of anaphylaxis, which may have reached epidemic levels in developed countries [4–8].

For example, Lee et al. reported an estimated annual anaphylactic incidence rate of 42 per 100,000 person-years from 2001 to 2010 in Olmsted County, Minnesota, U.S. [8]. In a U.K. study cohort, Gonzalez-Perez et al. documented that the incidence rate of anaphylaxis was 21 and 50 per 100,000 person-years among subjects with asthma and without asthma, respectively, from 1996 to 2005 [9]. In recognizing its rapidly increasing incidence, anaphylaxis has attracted substantial public health attention in developed countries during the past years. Due to its significant economic and healthcare burden, the anaphylaxis epidemic has led to increasing demand for specialty and medical services [10]. Nevertheless, the majority of epidemiologic studies on incidence of anaphylaxis have been from Western developed countries, and relatively few studies assess the incidence of anaphylaxis in Asian countries, including Taiwan [11].

In the present study, we aimed to investigate time trends in the incidence rate of anaphylaxis using a representative nationwide sample of an Asian population from Taiwan’s National Health Insurance Research Database (NHIRD) from 2001 to 2013, to examine the effect of age and gender on having an anaphylactic episode, and to evaluate the trends of severity of anaphylactic episodes in an Asian population.

## Methods

### Data source

The study cohort was three million subjects obtained from three different Longitudinal Health Insurance Databases (LHID) composed of medical claims data from the National Health Insurance Research Database (NHIRD) in Taiwan. The National Health Insurance (NHI) program has provided mandatory medical care to residents in Taiwan since 1995. The NHIRD derived from the medical reimbursement of the NHI program provided medical claims data, including demographic characteristics, disease diagnoses, ambulatory care and inpatient claims data, and prescription records. Currently, the NHI program covers enrollees representing nearly 98% of the total population in Taiwan [12]. Data used in this study were collected from three different LHID datasets. In detail, each LHID dataset used in this study was constructed by randomly selecting one million subjects from the NHI program in 2000, 2005, and 2010, individually. We included medical claims data of approximately three million subjects from January 1, 2001 to December 31, 2013 in this study. The Institutional Review Board of the National Health Research Institutes, Taiwan, approved this study protocol.

### Study cohort

**Fig. 1:**
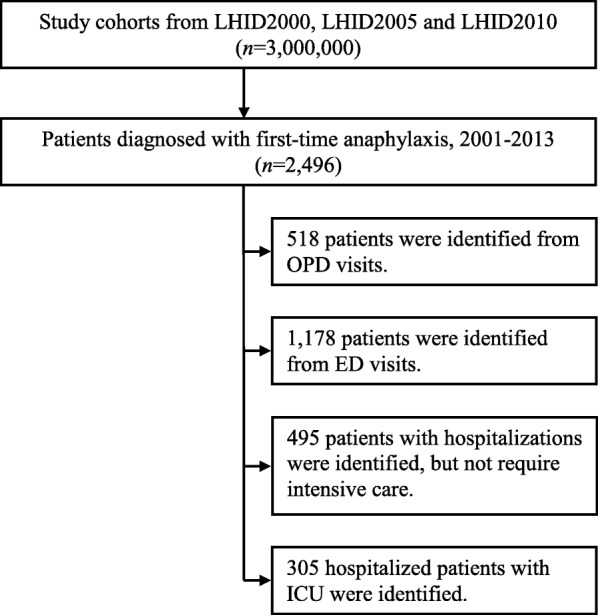
Flow chart showing the identification of patients with anaphylaxis. LHID: Longitudinal Health Insurance Database; OPD: outpatient department; ED: emergency department; ICU: intensive care unit. ICD-9-CM codes for anaphylaxis: 995.0, 995.60–995.69, 999.41–999.42, and 999.49)

During the 2001–2013 study period, we identified subjects with first-time anaphylaxis according to the International Classification of Diseases, 9th Revision, Clinical Modification [ICD-9-CM] diagnosis codes: 995.0, 995.60–995.69, 999.41–999.42, and 999.49 from outpatient department (OPD) or emergency department (ED) visits, or inpatient admissions, respectively, based on the World Allergy Organization anaphylaxis guidelines [13, 14]. The index date was defined as the first date of anaphylaxis diagnosis. Figure 1 depicts the detailed flow chart in relation to the identification of the study patients.

### Data analysis

The primary outcomes investigated in this study were anaphylaxis-related OPD visits, ED visits, hospitalizations, and admissions to an intensive care unit (ICU), separately. We calculated annual incidence rates of anaphylaxis-related events by dividing the number of new cases of anaphylaxis by the number of observed person-years. Annual incidence rate was expressed as the number of the first-time anaphylactic patients per 100,000 person-years for each study year. We performed Poisson regression models to evaluate the relationships of incidence rates with various age groups, gender, anaphylaxis-related OPD visits, ED visits, hospitalizations, and admissions to an ICU, respectively. We then applied linear regression analysis to examine time trends of anaphylaxis-related OPD visits, ED visits, hospitalizations, and admissions to an ICU, separately, across study years. All analyses were performed using SAS version 8.2 (SAS institute, Cary, NC). *P* values less than 0.05 were declared to be statistically significant.

## Results

Demographic characteristics of the 2496 incident cases of anaphylaxis are summarized in Table 1. The mean age of patients with first-time anaphylaxis was 45.11 years (standard deviation (SD), 20.64 years [range 3 months-100 years]), 56% (*n* = 1391) of patients were male. Nearly half of the cases (47%) were treated in the ED, while 21% (*n* = 518) were evaluated and managed in the OPD. Twenty percent were hospitalized, and 12% (*n* = 305) were further required admission to an ICU (Table 1). The distribution of urbanization among study patients was suburban area (48%), followed by urban area (24%), and rural area (11%), respectively (Table 1). A detailed flow chart related to identification of the study subjects is shown in Fig. 1.

**Table 1:** Demographic characteristics of study patients with anaphylaxis (*n* = 2496)

General characteristics	Number	Percent
Age (years)		
≤ 18	292	12
19–39	697	28
40–59	858	34
≥ 60	649	26
Gender		
Males	1391	56
Females	1105	44
Disposition		
Outpatient department	518	21
Emergency department	1178	47
Hospitalizations	495	20
Intensive care unit	305	12
Urbanization		
Urban	604	24
Suburban	1205	48
Rural	262	11
Unknown	425	17

**Fig. 2:**
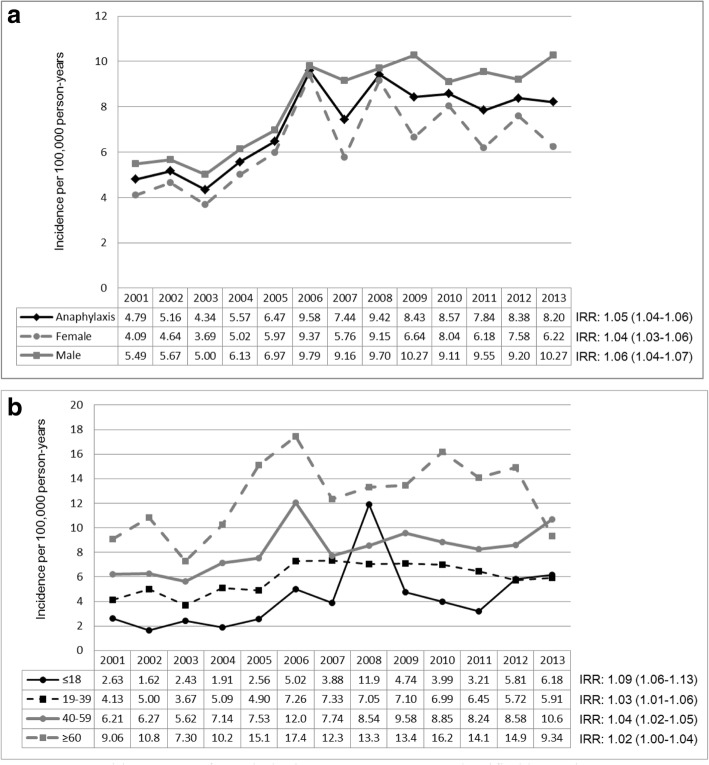
**a.** Incidence rate of anaphylaxis across 2001–2013, stratified by gender. **b.** Incidence rate of anaphylaxis across 2001–2013, classified by various age groups

The overall incidence of anaphylaxis was 7.25 (95%CI = 6.97–7.53) per 100,000 person-years. There was significant difference in incidence rates between children aged less than 18 years and adults aged 18 years or older (4.06 per 100,000 person-years, 95%CI = 3.60–4.53 in children; 8.1 per 100,000 person-years, 95% CI = 7.75–8.43 in adults; *p*__difference_ < 0.01). The incidence rate of anaphylaxis has increased from 4.79 per 100,000 person-years in 2001 to 8.20 per 100,000 person-years in 2013, with an incidence rate ratio (IRR) of 1.05 (95%CI = 1.04–1.06) (Fig. 2), indicating that there was a 5% annual increase.

As shown in Table 2, the incidence rate of anaphylaxis was significantly greater in males than in females (8.17 per 100,000 person-years, 95%CI = 7.74–8.60 in males; 6.35 person-years, 95%CI = 5.98–6.73 in females; *p* < 0.01). The incidence rate of anaphylaxis (Table 2) also increased with age in both genders (both *p* < 0.01). Figure 2a shows that the incidence rates of anaphylaxis in both males and females (IRR = 1.06, 95%CI = 1.04–1.07; *p*__trend_ < 0.01 in males; IRR = 1.04, 95%CI = 1.03–1.06; *p*__trend_ < 0.01 in females) increased significantly over time (2001–2013).

**Table 2:** Incidence of anaphylaxis in Taiwan, 2001–2013, by gender and various age groups

	Male		Female		Total	
	n	Rate^*^	n	Rate^*^	n	Rate^*^
Age at diagnosis (years)						
≤ 18	155	4.17	137	3.95	292	4.06
19–39	412	7.19	285	4.64	697	5.87
40–59	495	9.69	363	7.01	858	8.34
≥ 60	329	13.32	320	12.24	649	12.77
Total	1391	8.17	1105	6.35	2496	7.25
*P*-value^&^	*P*__gender_ < 0.01					
*P*-value^†^		*P*__age_male_ < 0.01		*P*__age_female_ < 0.01		*P*__age_total_ < 0.01

^*^ Incidence per 100,000 person-years

^&^*P*-value for differences in incidence rates among gender (2001–2013)

^†^*P*-value for differences in incidence rates among age groups (2001–2013)

The incidence rates of anaphylaxis varied by age groups as shown in Fig. 2b. The overall incidence rates of anaphylaxis were 4.06 (95%CI = 3.60–4.53) per 100,000 person-years in subjects ages ≤ 18 years; 5.87 (95%CI = 5.44–6.31) per 100,000 person-years in subjects ages 19–39 years; 8.34 (95%CI = 7.78–8.90) per 100,000 person-years in subjects ages 40–59 years; and 12.77 (95%CI = 11.78–13.75) per 100,000 person-years in subjects ages ≥ 60 years. When we evaluated the trends of incidence rates in each age group, the incidence rates of anaphylaxis significantly increased in subjects ages ≤ 18 years (incidence rate ratio (IRR) = 1.09, 95%CI = 1.06–1.13; *p*__trend_ < 0.01), 19–39 years (IRR = 1.03, 95%CI = 1.01–1.06; *p*__trend_ < 0.01), and 40–59 years (IRR = 1.04, 95%CI = 1.02–1.05; *p*__trend_ < 0.01), respectively, but not in subjects ages ≥ 60 years (IRR = 1.02, 95%CI = 1.00–1.04; *p*__trend_ = 0.05).

**Fig. 3:**
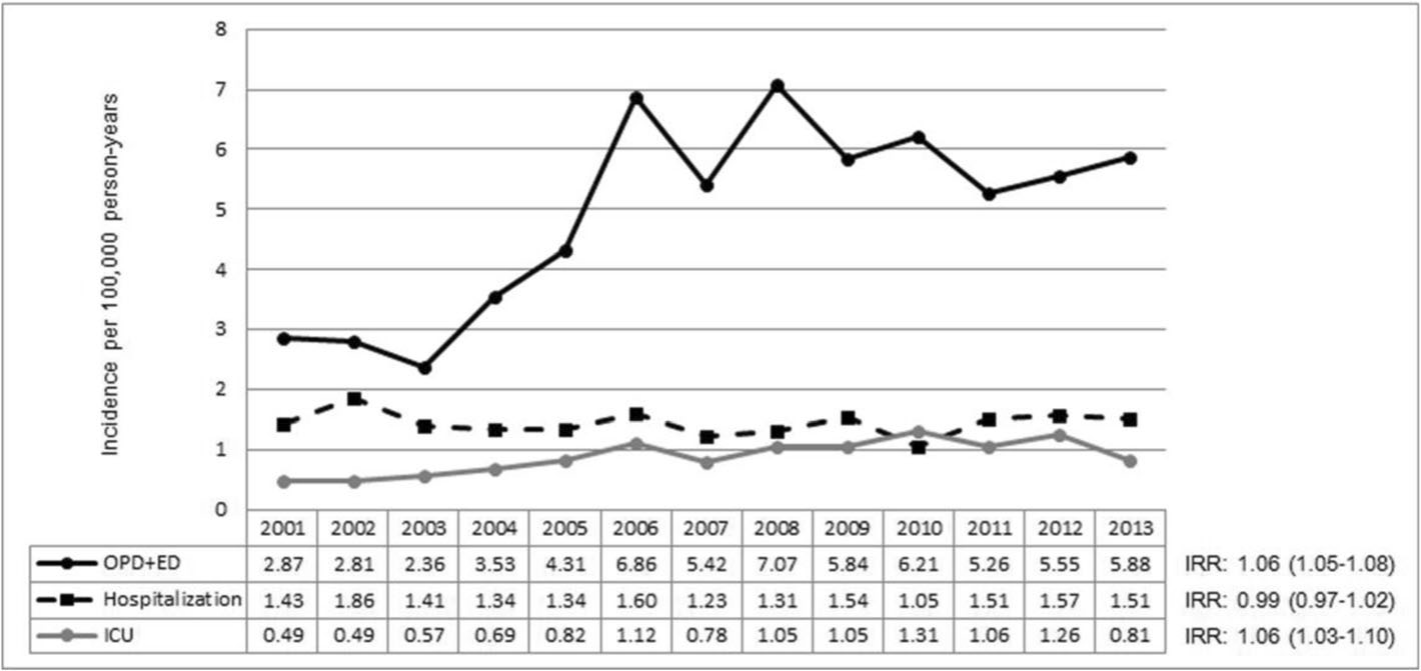
Time trends in incidence rates of outpatient department or emergency department visits, admissions to an inpatient ward, and admissions to an intensive care unit, for anaphylaxis across 2001–2013. OPD: outpatient department; ED: emergency department; ICU: intensive care unit

**Fig. 4:**
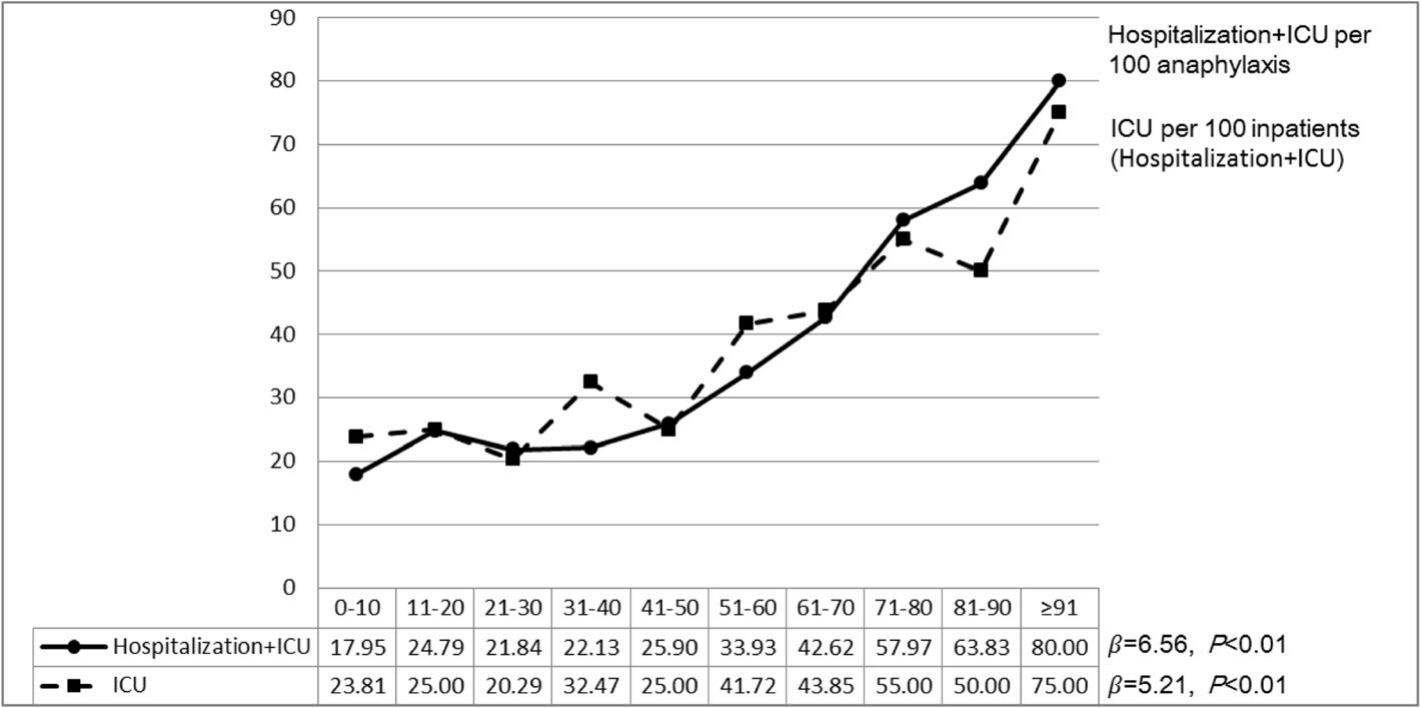
Proportions of patients with anaphylaxis admitted to the hospital (either inpatient ward or intensive care unit) or an intensive care unit across age groups. ICU: intensive care unit

Figure 3 shows the time trends in anaphylaxis-related incidence rates of OPD or ED visits, hospitalizations, and admissions to an ICU, separately, across 2001–2013. The time trends in the incidence of anaphylaxis-related OPD or ED visits have increased (IRR = 1.06, 95%CI = 1.05–1.08; *p*__trend_ < 0.01). The time trends in the incidence of anaphylaxis-related admissions to an ICU have also increased (IRR = 1.06, 95%CI = 1.03–1.10; *p*__trend_ < 0.01), whereas hospitalizations have remained steady over time (IRR = 0.99, 95% CI = 0.97–1.02; *p*__trend_ = 0.66). In addition, the proportion of patients requiring hospitalizations including ICU among all patients (*β* = 6.56; *p*__trend_ = 0.01), and the proportion requiring intensive care treatment among hospitalized patients (*β* = 5.21; *p*__trend_ = 0.01) increased with age (Fig. 4 and Additional file 1: Table S1).

## Discussion

To date, limited studies related to incidence rate of anaphylaxis have been reported in Asian populations. This is one of the largest nationwide, population-based studies investigating long-term time trends in the incidence rate of anaphylaxis in an Asian population. The overall incidence rate of anaphylaxis was 7.25 per 100,000 person-years from 2001 to 2013, with the incidence rate being 8.1 and 4.06 per 100,000 person-years in adults and children, respectively. The incidence rate increased from 4.79 per 100,000 person-years in 2001 to 8.20 per 100,000 person-years in 2013, with an incidence rate ratio of 1.05 (95% CI = 1.04–1.06), indicating that there was a 5% annual increase in the incidence rate of anaphylaxis. We identified a significant rise in incidence rates of anaphylaxis in both genders over the 13-year period, mainly in subjects aged less than 60 years, with the most prominent increasing trend observed in children aged less than 18 years. The proportion of patients requiring hospitalizations or ICU among all patients with anaphylaxis increased with age.

The steady increase (5% increase per year) in the anaphylaxis-related incidence rates in the current study is similar to those previously reported in the U.S., the U.K., Australia, and Spain [8, 15, 16]. In accordance with the national estimates in this study, Hsin et al. observed an increase in incidence of anaphylaxis from 4.7 per 100,000 patients in 2002 to 12.8 per 100,000 patients in 2010 in a single hospital-based study in Taiwan [17]. Similar to our observation in Taiwan, Yang et al. reported a 2-fold increase in the incidence of anaphylaxis from 16.02 per 100,000 person-years in 2008 to 32.19 per 100,000 person-years in 2014 in Korea [18]. While our results are consistent with the results of Hsin et al. and Yang et al., our study includes a significantly larger population.

Explanations for the increasing anaphylactic incidence rates in Taiwan remain speculative but could be mirroring the increasing prevalence of other allergic diseases, such as allergic rhinitis and food allergy, or could be secondary to increased diagnosis of anaphylactic episodes, or both [19]. The time trend observed in this nationwide study in Taiwan is comparable with previous incidence estimates in Western developed countries, suggesting that the increasing incidence of anaphylaxis is not only observed in Western countries but also in Asia. The cause of the recent increases in the incidence of anaphylaxis around the world remains unclear. Potential explanations may be lifestyle changes, differential exposure to environmental risk factors, and inherited epigenetic changes activated or suppressed by genes pertinent to immune regulation [20, 21]. Further studies are merited in delineating the underlying responsible mechanisms.

Previous epidemiological studies utilizing electronic databases to obtain the incidence rates of anaphylaxis are summarized in Additional file 1: Figure S1 [4, 6–9, 22–27]. The results show that anaphylaxis-related incidence varies across different countries, ranging from 6.7 to 112.2 per 100,000 person-years (Additional file 1: Figure S1). Despite consistent trend in elevated incidence of anaphylaxis, the estimated incidence in our study population is relatively low compared to the estimated incidence in Western developed countries [4, 5, 7–9, 26]. The observation may be partially explained by environmental variation and/or genetic difference. Previous studies have indicated that food allergies trigger the majority of anaphylactic episodes; among food specific allergens, peanuts and tree nuts are the main sources responsible for anaphylactic adverse events [28]. In parallel, hospitalizations due to food-induced anaphylaxis have elevated more than 3-time during the past years in the U.S. and U.K. [7, 29]. It has been noted that the prevalence of peanut allergy in Taiwan is lower than that reported in Western countries, which may account for the observed relatively low anaphylaxis-related incidence in this study [30]. It is also probably that the relatively low anaphylactic incidence may be due to difference in genetic background across different ethnicities.

Few population-based studies have documented that the occurrence of anaphylaxis varies by age and gender [31–34]. For example, Rudders et al. reported age-related differences in food-induced anaphylaxis and underscored the necessity for improving awareness of food-induced anaphylaxis in pediatric populations [33]. In addition, previous reports indicate that common triggers of anaphylaxis are different between children and adults [32]. Similarly, gender differences in anaphylactic incidence have remained unclear [31]. Our study found that incidence of anaphylaxis increased as age increased and had a male predominance. To date, age and/or gender differences in incidence of anaphylaxis are largely unexplored. Therefore, the observed results should be interpreted with caution and is warranted for further investigation.

This study demonstrates a clear increase in the proportion of patients with anaphylaxis requiring hospitalizations and/or an ICU as age increases. Likewise, the rising proportion of patients requiring intensive care treatment among those hospitalized was also observed as age increased. Our results are consistent with previous studies [35, 36]. For example, Jeppesen et al. have found an increase in hospitalizations due to anaphylaxis in all ages in Denmark [35]. The observed rising rate of anaphylaxis related hospitalization raises some concern whether anaphylactic episodes have gotten more severe over the past years. In addition, our results indicated an increasing proportion of hospitalized patients requires admission to the ICU, which is barely explored. It will be of importance to further validate the findings in this study.

The major strengths of this study are that we used national medical claims databases, which allow us to provide a nationwide estimated incidence of anaphylaxis over a 13-year period in a Taiwanese population. In addition, a large population-based sample from the NHIRD allows us to evaluate the trends of management administered in Taiwan’s medical facilities over the study period. Moreover, selection bias or information bias may not be a concern because of nationwide sampling. Nevertheless, this study has some limitations. First, similar to most studies using medical claims data, our data lack detailed information regarding specific inciting triggers; however, such inaccuracies probably remained unchanged between 2001 and 2013. Nonetheless, we found that the most common triggers among patients with first-time anaphylaxis are foods, approximately 23.5%, in this study. Second, this single-country study was conducted in Taiwan, which may or may not be generalizable to other Asian countries. Future efforts are needed to understand the time trends in the anaphylactic incidence in other Asian countries. Third, we cannot exclude the potential bias caused by unmeasured confounding.

In summary, this study provides the national estimates of the incidence of anaphylaxis in Taiwan’s general population over a 13-year period, 2001–2013. The steady increase in the incidence rate of anaphylaxis in both genders and most strikingly in children less than 18 years of age in Taiwan is consistent with those previously reported in Western countries. Our findings suggest that the increasing incidence of anaphylaxis is a public health threat, not only affecting the Western populations, but also Asian populations. Our results highlight the necessity of disentangling the potential reasons responsible for the rising incidence of anaphylaxis in Taiwan and other countries.

## Additional file

Additional file 1: **Table S1.** Management of first-time anaphylaxis in Taiwan, 2001–2013. **Figure S1.** Incidence rate of anaphylaxis reported from different countries. (DOCX 19 kb)

## Abbreviations

CI: confidence interval
ED: emergency department
ICD-9-CM: International Classification of Diseases, 9th Revision, Clinical Modification
ICU: intensive care unit
IRR: incidence rate ratio
LHID: Longitudinal Health Insurance Database
NHI: National Health Insurance
NHIRD: National Health Insurance Research Database
OPD: outpatient department
SD: standard deviation
