# Supplementary material for: Increasing trends of anaphylaxis-related events: an analysis of anaphylaxis using nationwide data in Taiwan, 2001–2013
Source: World Allergy Organ J. 2018 Oct 10;11(1):23. doi: 10.1186/s40413-018-0202-7 (PMC6178262; doi:10.1186/s40413-018-0202-7)
Supplement: Supplementary file 1 — Table S1. Management of first-time anaphylaxis in Taiwan, 2001–2013. Figure S1. Incidence rate of anaphylaxis reported from different countries. (DOCX 19 kb) [file 40413_2018_202_MOESM1_ESM.docx]

**Table S1.** Management of first-time anaphylaxis in Taiwan, 2001-2013.

| **Age** (year) | **OPD**, *n* (%) | **ED**, *n* (%) | **Hospitalizations**,  *n* (%) | **ICU**, *n* (%) | **All patients**, *n* (%) |
| --- | --- | --- | --- | --- | --- |
| **≤18** | 110 (37.67) | 115 (39.38) | 48 (16.44) | 19 (6.51) | 292 (100.00) |
| **19-39** | 147 (21.09) | 400 (57.39) | 114 (16.36) | 36 (5.16) | 697 (100.00) |
| **40-59** | 166 (19.35) | 431 (50.23) | 172 (20.05) | 89 (10.37) | 858 (100.00) |
| **≥60** | 95 (14.64) | 232 (35.75) | 161 (24.81) | 161 (24.81) | 649 (100.00) |
| **Total** | 518 (20.75) | 1,178 (47.20) | 495 (19.83) | 305 (12.22) | 2,496 (100.00) |

Abbreviation: **OPD:** outpatient department; **ED:** emergency department; **ICU:** intensive care unit.

**Figure S1.** Incidence rate of anaphylaxis reported from different countries.
